# The Variants Within the COL5A1 Gene are Associated with Reduced Risk of Anterior Cruciate Ligament Injury in Skiers

**DOI:** 10.1515/hukin-2015-0011

**Published:** 2015-04-07

**Authors:** Marta Stępień-Słodkowska, Krzysztof Ficek, Mariusz Kaczmarczyk, Agnieszka Maciejewska-Karłowska, Marek Sawczuk, Agata Leońska-Duniec, Miłosz Stępiński, Paweł Ziętek, Paweł Król, Monika Chudecka, Paweł Cięszczyk

**Affiliations:** 1 University of Szczecin, Department of Physical Culture and Health Promotion, Szczecin, Poland.; 2 Gdansk University of Physical Education and Sport, Faculty of Tourism and Recreation, Gdansk, Poland.; 3 Medical University at Szczecin, Department of Orthopedics and Traumatology of Pomeranian Medical, Szczecin, Poland.; 4 University of Rzeszow, Department of Physical Culture, Rzeszow, Poland.

**Keywords:** ACL rupture, collagen, polymorphisms, real-time polymerase chain reaction

## Abstract

The purpose of this study was to examine the association of the BstUI RFLP C/T (rs 12722) and DpnII RFLP C/T (rs 13946) COL5A1 polymorphisms, individually and as haplotypes, with anterior cruciate ligament ruptures in recreational skiers. Subjects were 138 male recreational skiers with surgically diagnosed primary anterior cruciate ligament ruptures. The control group consisted of 183 apparently healthy male recreational skiers, who were without any self-reported history of ligament or tendon injury. DNA was extracted from buccal cells donated by the subjects and genotyping was carried out using real-time PCR. The genotype distributions for both polymorphisms met Hardy-Weinberg expectations in both groups. There were no significant differences in genotype distribution of allele frequencies of COL5A1 BstUI RFLP C/T and COL5A1 DpnII RFLP C/T polymorphisms between the ACL rupture and control groups. The T-T (BstUI RFLP T, DpnII RFLP T) haplotype was the most common (55.6%). The haplotype T-C was not present in any of the subjects. There was an underrepresentation tendency of the C-T haplotype in the study group compared to controls under recessive mode of inheritance. Higher frequency of the COL5A1 BstUI RFLP C/T and COL5A1DpnII RFLP C/T polymorphisms haplotype is associated with reduced risk of anterior cruciate ligament injury in a group of apparently healthy male recreational skiers.

## Introduction

Skiing as a recreational activity has gained a lot of interest in the last twenty years (O’Donnel et al., 2007). Injuries in alpine skiing have been a serious concern since the very beginning of the sport (Spörri et al., 2012). The injury pattern has changed over this time period, due to alterations and improvements in ski equipment (O’Donnel et al., 2007), however, the risk of injury is still high. During a single skiing season throughout Switzerland, around 1000 alpine skiers and snowboarders per day were diagnosed with injuries serious enough to require medical treatment (Hasler et al., 2011). The knees are most commonly affected. Injuries to the anterior cruciate ligament of the knee are common in populations with regular physical activity and may seriously influence the risk of early-onset posttraumatic osteoarthritis regardless of the treatment administered (Smith et al., 2011). Recent developments that enhance understanding of the role of individual genes in human body’s processes should contribute to injury prevention as well as sport rehabilitation. Thus, the role of genetics in sport research increases each year (Sawczuk et al., 2011).

The knee joint comprises a number of tissues: bones, muscle, tendons and ligaments, among others (Boyan et al., 2013). It relies on other structures to provide both static and dynamic stability: the anterior and posterior cruciate ligaments, the medial and lateral collateral ligaments, the menisci, the capsule and the muscles crossing the joint (Frizziero et al., 2013). Knee stability is largely dependent on the ability of the anterior cruciate ligament (ACL) to resist anterior tibial translational loads and rotational loads (Dhillon et al., 2011).

Most ACL injuries in sports occur during a non-contact episode, typically during deceleration, lateral pivoting, or landing tasks that are associated with high loads on the knee joint. Therefore, sports maneuvers may lead to high knee loads (Myer et al., 2011). The most frequent “slip-catch” mechanism of ACL injury was identified in the World Cup alpine skiing. The injury mechanism is characterized by a common pattern in which the inside edge of the outer ski catches the snow surface while turning, forcing the knee into valgus and tibial internal rotation (Bere et al., 2013). However, numerous studies have shown that ACL ruptures are complex, multifactorial and determined by the interaction of extrinsic and intrinsic risk factors (Hewett et al., 2010).

The ACL is composed of dense fibrous connective tissue that attaches the femur to the tibia. It has a large amount of collagen fibers arranged in a hierarchal pattern giving it high tensile strength (Tovar et al., 2010). Collagen is the main component of ligaments (O’Connell et al., 2013) and its fibrils may be composed of collagen type I, III, VI, V, XI and XIV (Frank et al., 2004). Type V collagen is a quantitatively minor fibrillar collagen, but most prominent in connective tissue where it plays an important role in regulating fiber diameter, initiating fibril assembly and regulating lateral fibril growth within tendons and ligaments (Posthumus et al., 2011b; Brown et al., 2011b). Collagen type V is a protein encoded by the COL5A1 gene which is located on the long (q) arm of chromosome 9 (Collins and Posthumus, 2011). Mutations within the COL5A1 gene cause the classic form of Ehlers-Danlos syndrome characterized by joint hyper mobility (Brown et al., 2011) and other joint dysfunctions (Collins and Posthumus, 2011). Therefore, our attention was directed toward COL5A1 sequence variants as potential intrinsic risk factors for the ACL rupture.

The COL5A1 gene contains BstUI and DpnII restriction fragment length polymorphisms (RFLPs) within its 3′-untranslated region (UTR) (Greenspan and Pasquinelli, 1994). The functional COL5A1 Sp1 binding site polymorphism - BstUI RFLP, known also as: rs 12722, C/T is associated with a number of sport injuries. This polymorphism was detected in a physically active Caucasian population in South Africa (Posthumus et al., 2010).

The CC genotype of this variant was overrepresented significantly in the asymptomatic control participants of the study. The CC genotype of SNP rs 12722 was also overrepresented in the asymptomatic control participants among a physically active Caucasian population in Australia. The individuals with a wild-type CC genotype are protected against chronic degenerative changes in the Achilles tendon during running and other forms of physical activity (Collins and Posthumus, 2011). Similar results were reported when female participants with ACL ruptures were compared with asymptomatic female controls. This data suggests that individuals with a CC genotype are protected, despite the load or external forces applied to their musculoskeletal soft tissues. Another polymorphism which we investigated was DpnII RFLP, known also as rs 13946, C/T within the COL5A1 gene. A Mokone et al. (2006) study revealed a significant difference in the allele frequencies of the COL5A1 BstUI RFLP between the Achilles tendon pathology group and control subjects. However, the influence of both polymorphisms on the expression of the COL5A1 gene is not completely known.

The aim of this study was to examine the association of the BstUI RFLP and DpnII RFLP polymorphisms in the COL5A1 gene, individually and as haplotypes, with ACL ruptures in recreational skiers. We postulated that the COL5A1 BstUI RFLP and COL5A1 DpnII RFLP polymorphisms would be individually associated with the ACL rupture and that interaction between the COL5A1 BstUI RFLP and COL5A1 DpnII RFLP polymorphisms as a haplotype would predispose recreational skiers to higher risk of the ACL rupture.

## Material and Methods

The Pomeranian Medical University Ethics Committee approved the study and written informed consent was obtained from each participant. The study complied with the guidelines set out in the ethics policy of the Szczecin University (Kruk, 2013).

A total of 138 male recreational skiers (27 ±2 years) with surgically diagnosed primary ACL ruptures were recruited for this study, all of whom qualified for ligament reconstruction. The control group was comprised of 183 apparently healthy male skiers (26 ±3 years) with a comparable level of exposure to ACL injury, none of whom had any self-reported history of ligament or tendon injury.

We followed recent recommendations for genotype-phenotype association studies (Chanock et al., 2007; Attia et al., 2009). Genomic DNA was extracted from the oral epithelial cells using GenElute Mammalian Genomic DNA Miniprep Kit (Sigma, Germany) according to the manufacturer’s protocol. Allelic discrimination of *COL5A1* BstUI RFLP C/T (rs 12722) and DpnII RFLP C/T (rs 13946) polymorphic sites was performed using a TaqMan Pre-Designed SNP Genotyping Assays (Applied Biosystems, USA), including primers and fluorescently labelled (FAM and VIC) probes for the detection of alleles. All samples were genotyped on a real-time polymerase chain reaction (PCR) instrument (StepOne™, Applied Biosystems, USA). Thermal cycler conditions were as follows: an initial step at 95°C for 5 min, followed by 45 cycles of denaturation at 94°C for 15 s and anneal/extend at 60°C for 1 min. We used positive and negative controls for the detection of both polymorphisms. The results were gathered by two experienced and independent investigators who were blind to the participants’ data.

### Statistical analysis

Some differences in the genotypes and allele frequency were analyzed using 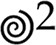 or Fisher exact tests. Allelic-based odds ratios (OR) with 95% confidence intervals (95% CI) were calculated using logistic regression analysis. A post hoc power calculation was detected for each gene variant. The genotypes between cases and controls were compared in three ways. First, a general test of association in the 2-by-3 table of phenotype-by-genotype was performed. Then two different modes of inheritance of minor allele were assumed: dominant, in which homozygotes and heterozygotes for the minor allele were pooled and compared to homozygotes for the major allele, and recessive, in which homozygotes for the minor allele were compared to pooled homozygotes and heterozygotes for the major allele. In addition, the programming language and environment R (http://www.r-project.org) were used for Hardy-Weinberg, linkage disequilibrium (LD) testing (package genetics), for the haplotype analysis (package haplo.stats). The haplo.em, haplo.group, and haplo.score functions of the haplo.stats package were used to infer haplotype frequencies and to test the association between reconstructed haplotypes and the risk of the ACL rupture assuming three possible haplotype effects: additive, dominant and recessive. Hap.score is the statistical score for haplotypes reflecting the strength of association; the positive value of hap.score indicates increased risk of ACL injury for a particular haplotype, while the negative value indicates reduced risk. The pair-wise linkage disequilibrium between – BstUI RFLP C/T and DpnII RFLP C/T was estimated by D′ and r^2^. For all tests, the level of significance was set at p<0.05.

## Results

At baseline, male recreational skiers with surgically diagnosed primary ACL ruptures and the control group did not differ significantly by age (27 ±2 and 26 ±3 years, respectively), and the level of exposure to ACL injury. The genotype and allele frequencies for the COL5A1 BstUI RFLP C/T (rs12722) and COL5A1 DpnII RFLP C/T (rs13946) are shown in Table 1. The genotype distributions for both polymorphisms met Hardy-Weinberg expectations in both groups.

There were no significant differences in genotype distribution or allele frequency of either COL5A1 BstUI RFLP (rs12722, C/T) or COL5A1 DpnII RFLP (rs13946, C/T) polymorphisms between the ACL rupture group and the control group using the 2-by-3 general test of association (Table 1). Likewise, there were no significant differences in the genotype frequencies for the COL5A1 BstUI RFLP C/T and DpnII RFLP C/T polymorphisms, when dominant and recessive modes of inheritance were assumed.

COL5A1 BstUI RFLP C/T and DpnII RFLP C/T were found to be in linkage disequilibrium (Table 2). A total of three reconstituted haplotypes with estimated frequency > 0.05 were found, and only those were evaluated for an association with the ACL rupture. The T-T (BstUI RFLP T, DpnII RFLP T) haplotype was the most common (55.5%). The haplotype T-C was not present in any of the subjects.

Then, we tested the association between these haplotypes and the ACL rupture assuming three haplotype effects; a) additive – considering the count of a particular haplotype as 0, 1, 2, b) dominant – heterozygous or homozygous carrier of a particular haplotype versus otherwise, c) recessive – homozygous for a particular haplotype versus otherwise. None of these yielded statistically significant results, however there was an underrepresentation tendency of the C-T haplotype in the ACLR group compared to controls under the recessive mode of inheritance (Table 3).

## Discussion

The biological mechanisms involved in non-contact musculoskeletal soft tissue injuries are poorly understood, but genetic risk factors may be associated with susceptibility to injuries. A single nucleotide polymorphism - DNA sequence variation can occur throughout the genome and can also affect the response of an individual to a specific treatment or other stimuli (Pruna et al., 2013). Smith et al. (2012) reported familiar predisposition to non-contact ACL tears. The genetic influence was assessed among others with a medical history questionnaire to determine the knee ligament injuries of the patients’ primary family members. There was a higher incidence rate of ACL injury in the immediate family of the injured group (35%) than that of the control group (4%).

In our study, we examined the association between both BstUI RFLP C/T (rs12722) and DpnII RFLP C/T (rs13946) polymorphisms in the *COL5A1* gene, individually and as haplotypes, with anterior cruciate ligament ruptures in recreational Polish skiers. Our findings showed no significant differences in genotype distribution or allele frequencies of *COL5A1* BstUI RFLP C/T and *COL5A1* DpnII RFLP C/T polymorphisms between the ACL rupture group and control group. Thus, our initial hypothesis was not confirmed. However, we found an underrepresentation tendency of the C-T haplotype in the ACLR group compared to controls (p=0.091). Higher frequency of the *COL5A1* C-T (BstUI RFLP C/T and DpnII RFLP C/T polymorphisms) haplotype among a group of apparently healthy male recreational skiers suggests an association with reduced risk of anterior cruciate ligament injury.

The results of the Smith et al. (2012) study helped to identify genetic factors associated with ACL tears. This study was based on unmatched case-control designs and was performed on a Caucasian South African population. The *COL1A1* gene encodes a protein chain within type I collagen, a major structural component of ligaments. A rare TT genotype of the *COL1A1* Sp1 binding site polymorphism was underrepresented in those with ACL injuries in comparison with the controls. ACL-injured subjects were 4 times more likely to have a family member with a ligament injury of any kind in comparison with controls. Another *COL12A1* gene encodes for protein chains in type XII collagen, which is believed to regulate fibril diameter in ligaments. The researchers found that the AA genotype of the *COL12A1* AluI polymorphism was overrepresented in female subjects with ACL injury. Recently, an association between the chromosomal region 11q22 and risk of the ACL tear was reported (Posthumus et al., 2012). Several matrix metalloproteinase genes, including physiologic mediators of collagen cleavage and removal, are located on the chromosome 11q22. In ACL-injured subjects, AG and GG genotypes of 1 matrix metalloproteinase variant were significantly underrepresented when compared to uninjured subjects. The frequency of these variant haplotypes within the gene was significantly different between injured and uninjured groups.

Likewise, the CC genotype of a variant in the *COL5A1* gene has been associated with ACL tears in females (Collins and Posthumus, 2011). The CC genotype was overrepresented in the asymptomatic control participants among a physically active Caucasian population in Australia. On the other hand, individuals with a wild-type CC genotype are protected against chronic degenerative changes in the Achilles tendon during running and other physical activity (O’Connell et al., 2013). O’Connell et al. (2013) support the hypothesis that variants within genes, such as *COL5A1*, *COL3A1*, *COL6A1*, and *COL12A1* genes that code for connective tissue components of the musculoskeletal system, may modulate susceptibility to exercise-associated muscle cramping (EAMC). The aim of mentioned study was to investigate a potential association between *COL5A1* rs12722 (C/T), *COL3A1* rs1800255 (G/A), *COL6A1* rs35796750 (T/C), and *COL12A1* rs970547 (A/G) polymorphisms and a history of EAMC. The *COL5A1* CC genotype was significantly overrepresented among the group without EAMC (22%) when compared with the EAMC group (11%). Thus, the *COL5A1* gene as a potential marker for a history of EAMC was identified for the first time. The *COL5A1* (the variant rs12722, BstUI RFLP) may also be a candidate gene associated with the development of the bilateral quadriceps tendon rupture (Longo et al., 2010). A polymorphism (rs12722) within the functional *COL5A1* 3′-untranslated region (UTR) has been shown to associate with chronic Achilles tendinopathy and other exercise-related phenotypes (Abrahams et al., 2013). This investigation included over 600 Caucasians participants who were genotyped for the *COL5A1* 3′-UTR markers rs71746744, rs16399, rs1134170 and marker rs4919510 within MIR608. All genetic markers were independently associated with chronic Achilles tendinopathy.

Studies by Raleigh et al. (2009) showed variants within the MMP3 gene were associated with Achilles tendinopathy. There were significant associations between the GG genotype of rs679620, the CC genotype of rs591058, the AA genotype of rs650108 and risk of Achilles tendinopathy. The ATG haplotype (rs679620, rs591058, and rs650108) was underrepresented in the tendinopathy group when compared to the control group.

A strength of our study is the homogeneity of the investigated groups. However, this fact may also be considered as a limitation, because only male skiers were examined. Other studies suggest that the rate of the ACL rupture is even three times higher in female than in male athletes (Warden et al., 2006). Female athletes landing with inadequate knee flexion, in greater-than-normal valgus, and external rotation are at increased risk of ACL injury when compared to men participating in similar athletic activities (Hewett et al., 2010; Sutton, 2013). Intrinsic factors such as an increased quadriceps angle, an increased posterior tibial slope, and estrogen fluctuation may also predispose females to ACL injury (Warden et al., 2006).

The importance of genetics in sport increases every year. Studies of the genetic factors that influence the risk of injuries are still scarce, even though they may provide a useful tool for the sports selection process, individualization of the training programs or sports traumatology. The results of the present study indicate that modifying training loads or physical therapy may be used as preventive means in athletes that are genetically predisposed to ACL injury.

## Conclusions for competitive sports

The findings of the present study provide a better understanding of which genetic profiles contribute to higher ACL injury risk.

We indicated that there was an underrepresentation tendency of the C-T haplotype in the ACLR group when compared to controls (p=0.091). Higher frequency of the *COL5A1* C-T (BstUI RFLP C/T and DpnII RFLP C/T polymorphisms) haplotype among a group of apparently healthy male skiers suggests an association with reduced risk of anterior cruciate ligament injury.

Analysis of the complex relationship between gene variants and ACL ruptures among athletes may assist clinicians and athletes to optimize training and to reduce the risk of the ACL rupture.

## Figures and Tables

**Table 1:** Association between the presence of either COL5A1 BstUI RFLP (rs12722, C/T) or COL5A1 DpnII RFLP (rs13946, C/T) polymorphisms and incidence of ACL rupture

SNP	Group	HWE	Genotype distribution	*p*	Allele frequency	*p*	OR (95%CI)
BstUI RFLPC/T	ACLR n = 138	0.861	CC: 24 (17%) CT: 66 (48%) TT: 48 (35%)	p 0.467 p_D_ 0.276 p_R_ 0.398	T: 59% C: 41%	0.218	0.82 (0.59–1.14)
	Control n = 183	1	CC: 39 (21%) CT: 91 (50%) TT: 53 (29%)		T: 54% C: 46%		

DpnII RFLP C/T	ACLR n = 138	1	CC: 12 (9%) CT: 57 (41%) TT: 69 (50%)	p 0.393 p_D_ 0.499 p_R_ 0.387	T: 71% C: 29%	0.846	0.97 (0.68–1.38)
	Control n = 183	0.077	CC: 11 (6%) CT: 88(48%)		T: 70% C: 30% TT: 84 (46%)		

p values correspond to genotype distribution and allele frequency.

OR correspond to the odds ratio for the incidence of ACL rupture.

ACLR: anterior cruciate ligament rupture; HWE: Hardy–Weinberg Equilibrium;

OR: odds ratio; CI: confident interval, p_D_ and p_R_ are two-sided Fisher’s exact test probabilities for dominant (CC+CT vs TT) and recessive (CC+CT vs TT) modes of inheritance of the minor allele (BstUI RFLP C/T, DpnII RFLP C/T), respectively.

**Table 2:** Pair-wise linkage disequilibrium (LD) and inferred haplotype frequencies for COL5A1 BstUI RFLP C/T and DpnII RFLP C/T

Gene	SNP1	SNP2	D 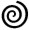	r^2^	Haplotypes	
					Haplotype	Frequency (%)
*COL5A1*	BstUI RFLP C/T (rs12722)	DpnII RFLP C/T (rs13946)	0.98^†^	0.52	T-T	56
					C-C	29
					C-T	15
					T-C	0.0
					Total	100.0

*D*′ *– the deviation of the observed frequency of a haplotype from the expected.*

*D*′*= 0.98 suggest on very high LD between COL5A1 BstUI RFLP C/T and COL5A1 DpnII RFLP C/T.*

Haplotypes are in decreasing frequency.

† p<0.0001

**Table 3:** Haplotypes frequencies in ACLR and controls (under a recessive mode of haplotype inheritance)

Haplotypes		Frequency (%)			Hap.score	*p*

BstUI RFLP C/T	DpnII RFLP C/T	Combined ACLR and controls	ACLR	Controls		
C	T	14.6	11.9	16.7	−1.689	0.091
C	C	29.3	29.3	29.3	−0.046	0.962
T	T	55.5	58.6	53.1	1.350	0.176

ACLR - anterior cruciate ligament rupture group;

Hap. score, statistics score for haplotypes.
